# Spatiotemporal Patterns of Reptile and Amphibian Road Fatalities in a Natura 2000 Area: A 12-Year Monitoring of the Lake Karla Mediterranean Wetland

**DOI:** 10.3390/ani14050708

**Published:** 2024-02-24

**Authors:** Alexandros D. Kouris, Apostolos Christopoulos, Konstantinos Vlachopoulos, Aikaterini Christopoulou, Panayiotis G. Dimitrakopoulos, Yiannis G. Zevgolis

**Affiliations:** 1 Biodiversity Conservation Laboratory, Department of Environment, University of the Aegean, 81132 Mytilene, Greece; alexkouris92@gmail.com (A.D.K.); pdimi@aegean.gr (P.G.D.); 2 Department of Zoology and Marine Biology, Faculty of Biology, National and Kapodistrian University of Athens, 15772 Athens, Greece; apochris@biol.uoa.gr; 3 Department of Agriculture, Crop Production and Rural Environment, University of Thessaly, 38446 Volos, Greece; kvlachopoulos@uth.gr; 4 Department of Economics and Sustainable Development, Harokopio University, 17676 Athens, Greece; achristopoulou@hua.gr

**Keywords:** herpetofauna roadkills, wetlands, protected areas, spatial statistics, KDE, Getis-Ord Gi*, KDE+, cluster strength, collective risk, Greece

## Abstract

**Simple Summary:**

The expansion of road networks poses a significant threat to wildlife, particularly reptiles and amphibians, within protected areas (PAs). To address this concern, we examined road mortality patterns among herpetofauna in a Greek-protected wetland over 12 years (2008–2019), utilizing a combination of statistical modeling and spatial analysis. We aimed to identify the most vulnerable species, seasonal variations, and ecological determinants of roadkill patterns. Across 14 documented species, 340 roadkill incidents were recorded, with snakes comprising over 60% of encounters. Both environmental and road-related factors significantly influenced roadkill risk. Spatial analysis techniques pinpointed critical hotspots, particularly in the southeastern region of the study area. These findings highlight the need for targeted mitigation strategies to protect herpetofauna within this PA. Understanding the specific factors influencing roadkill patterns is crucial for implementing effective conservation measures and safeguarding these vulnerable species.

**Abstract:**

The pervasive expansion of human-engineered infrastructure, particularly roads, has fundamentally reshaped landscapes, profoundly affecting wildlife interactions. Wildlife-vehicle collisions, a common consequence of this intricate interplay, frequently result in fatalities, extending their detrimental impact within Protected Areas (PAs). Among the faunal groups most susceptible to road mortality, reptiles and amphibians stand at the forefront, highlighting the urgent need for global comprehensive mitigation strategies. In Greece, where road infrastructure expansion has encroached upon a significant portion of the nation’s PAs, the plight of these road-vulnerable species demands immediate attention. To address this critical issue, we present a multifaceted and holistic approach to investigating and assessing the complex phenomenon of herpetofauna road mortality within the unique ecological context of the Lake Karla plain, a rehabilitated wetland complex within a PA. To unravel the intricacies of herpetofauna road mortality in the Lake Karla plain, we conducted a comprehensive 12-year investigation from 2008 to 2019. Employing a combination of statistical modeling and spatial analysis techniques, we aimed to identify the species most susceptible to these encounters, their temporal and seasonal variations, and the ecological determinants of their roadkill patterns. We documented a total of 340 roadkill incidents involving 14 herpetofauna species in the Lake Karla’s plain, with reptiles, particularly snakes, being more susceptible, accounting for over 60% of roadkill occurrences. Moreover, we found that environmental and road-related factors play a crucial role in influencing roadkill incidents, while spatial analysis techniques, including Kernel Density Estimation, the Getis-Ord Gi*, and the Kernel Density Estimation plus methods revealed critical areas, particularly in the south-eastern region of Lake Karla’s plain, offering guidance for targeted interventions to address both individual and collective risks associated with roadkill incidents.

## 1. Introduction

Dating back to 10,000 B.C., human-driven infrastructure has been integral to some of the earliest human constructions, contributing significantly to the growth of human economies and societies [1]. Since then, wherever humans have ventured, roads have inevitably followed, and where roads have been established, human settlements have emerged. The inevitable consequence of the continual expansion of transportation infrastructures is the encroachment into natural environments, leading to numerous adverse impacts on habitats and their inhabitants, who often find themselves unaware and unprepared, and have difficulty in coping [2,3].

The most evident impacts of roads on natural ecosystems, directly affecting habitats and indirectly influencing species, encompass pollution, both in water and air [4], modifications to hydrology and soil [5], and the facilitation of invasive alien species establishment [6,7,8]. Among these impacts, habitat fragmentation stands out as one of the most severe, transforming natural landscapes into fragmented patches, varying in size, interspersed with roads [9,10]. This phenomenon extends even into Protected Areas (PAs), as the majority of locations inhabited by humans are connected by some form of transportation infrastructure, contributing to their fragmentation [11].

In addition to the indirect impacts on ecosystems, roads directly affect faunal species, with collisions between vehicles and wildlife leading to severe injuries or fatalities. These incidents occur both outside of PAs [12], on road verges [13], and even within the boundaries of PAs [14,15]. The occurrence of roadkill and other associated effects within PAs is particularly alarming, as it contradicts the fundamental purpose of these areas—to conserve habitats, fauna, flora, and their biotic and abiotic functions [16]. This contradiction becomes more pronounced when considering that roadkill rates can be higher within the limits of PAs, possibly due to the increased diversity and abundance of wildlife inside these areas [14].

While roadkill is a phenomenon that can transpire in any natural habitat [17], affecting a broad spectrum of species from primates [18] to invertebrates [19], not all habitats and not all taxonomic groups are affected in the same way and at the same rate [20]. Reptiles and amphibians emerge as the groups most significantly impacted by road fatalities [21,22,23], with roadkill posing a paramount threat to the populations of these species [24]. This vulnerability stems from a synergistic interplay of physiological, behavioral, and ecological factors that converge to heighten their exposure to road hazards [25,26,27]. Physiological characteristics, such as inherent slowness and larger body sizes [28], particularly in snakes and turtles, significantly increase their susceptibility to vehicle collisions. Behavioral aspects further exacerbate the risk, as some species exhibit immobilization behavior in response to approaching vehicles, rendering them immobile and more vulnerable to collisions [29]. Additionally, some reptiles prolong their road usage for thermoregulation or to scavenge on carrion [30], increasing their exposure to road traffic. Ecological factors also play a critical role in driving reptiles and amphibians to cross roads, particularly during migration and breeding seasons. Migration, a crucial life stage, precipitates increased road crossings, with females and males often exhibiting distinct migratory patterns and risk profiles [23].

Road mortality exhibits variations not only among taxonomical groups and species but also across time and space. The season of the year and the time of the day can influence mortality rates differently [23,26]. Specifically, for reptiles and amphibians, the highest fatalities occur during summer and autumn, as these periods are characterized by elevated temperatures, crucial for thermoregulation, and favorable rainfall patterns that support migration [31,32,33]. In terms of the time of the day, it appears that nocturnal species experience fewer casualties compared to species active during the daytime [34].

In parallel, habitat plays a significant role, with several studies highlighting a strong connection between reptile and amphibian road fatalities and wet habitats, such as wetlands, compared to other ones like forests [35]. This association is rooted in the integral role wetlands play in the biology and ecology of both reptiles and amphibians [36,37]. For amphibians, wetlands serve as breeding grounds, support larval development, and act as the primary food source for adult amphibians, while for certain reptiles, wetlands function as their primary habitat, providing abundant food sources and suitable locations for breeding and nurturing offspring [38,39].

Case studies examining road fatalities near wetlands and lakes are notably scarce, with the United States providing significant examples [40,41,42] along with select European cases, including Austria [43], Italy [44], Slovenia [45], and Serbia [22]. However, the research in this specific context remains somewhat limited, leaving a noticeable gap in our understanding of the impacts of roads on fauna in these vital ecosystems. In contrast, a more extensive body of research delves into road mortality within or on the edges of PAs, spanning diverse regions globally. Investigations span from Australia [46] to South Africa [15], and from South America [47] to Canada [29]. Particularly in Europe and the Mediterranean, globally recognized as biodiversity hotspots, a multitude of studies dissect the impacts on reptiles and amphibians [13,14,44,48,49,50,51]. Nevertheless, this robust focus and exploration appear noticeably absent in Greece, unveiling a potential gap in our comprehension of the intricate challenges that fauna confront due to road-related issues in this area.

Despite Greece boasting a diverse herpetofauna with 26 amphibian and 69 reptile species, constituting 38% of all European reptiles and 27% of amphibians [52], the swift expansion of infrastructure, particularly road networks, poses a significant challenge to conservation efforts, threatening to fragment critical habitats and protected areas [53,54]. While the extensive Natura 2000 network provides a framework for conservation [55], significant gaps persist in understanding the specific impact of roads on fragmenting these sanctuaries, especially in sensitive wetland ecosystems and adjacent terrestrial zones, crucial for numerous herpetofauna species [56]. This knowledge deficiency hampers the development of effective mitigation strategies, leaving vulnerable herpetofauna populations exposed to the peril of roadkill incidents.

To fill this research gap, our study marks the inaugural endeavor to systematically document and analyze road fatalities of reptiles and amphibians in a protected, isolated wetland in Greece—specifically, Lake Karla. This wetland serves as a poignant example of a natural ecosystem sacrificed for arable land, illustrated by its drainage in 1962. By 2009, efforts had commenced to partially restore the lake, and a significant portion of Karla’s plain had become part of the Natura 2000 network.

This historical context sets the stage for our investigation, where we identified the herpetofauna species most vulnerable to road accidents and their spatiotemporal distribution patterns spanning from 2008 to 2019. Furthermore, we examined the wetlands’ road network, aiming to address multifaceted objectives: (a) to discern the most frequently road-killed herpetofauna species, (b) to scrutinize temporal and seasonal variations associated with these incidents, (c) to evaluate the intricate relationship between roadside habitats and road network characteristics by incorporating important environmental factors, (d) to predict the occurrence of reptile and amphibian roadkills, and (e) to identify their spatial distribution and hotspot areas.

## 2. Materials and Methods

### 2.1. Study Area

**Figure 1:**
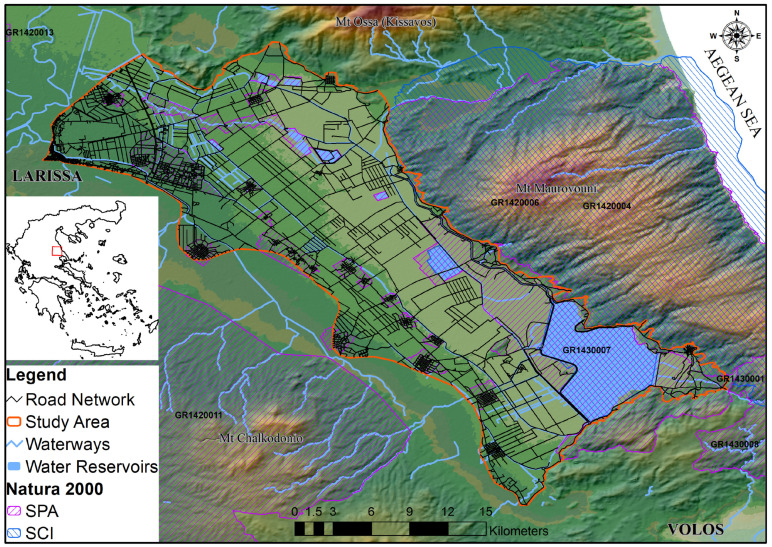
Lake Karla’s plain, showcasing the study area with the road network and the designated Natura 2000 areas. Noteworthy geographical features include Mount Ossa to the north, Mount Mavrovouni to the northeast, Mount Megavouni to the south, and Mount Chalcodoni to the southeast.

Lake Karla’s plain, situated in the eastern sector of the Thessaly region (39.49931° N, 22.77837° E), spans an approximate area of 480 km^2^. Historically, Lake Karla’s basin, once covering 1663 km^2^, played a pivotal role in water storage and groundwater recharge until its drainage in 1962. Since then, a phased restoration has begun, commencing with the southernmost part in 2009, facilitated by pumping stations and supply channels. Presently, it encompasses the restored Lake Karla, 13 reservoirs, and an artificial wetland (Figure 1). Dominated by freshwater aquatic plants, reedbeds, and sclerophyllous shrubs or phrygana, the area aligns with the Mediterranean climate, characterized by hot, dry summers and cold, wet winters [57]. A substantial portion of Lake Karla’s plain is presently incorporated within the Natura 2000 network with the establishment of two SPA (“Reservoirs of Former Lake Karla, GR1430007” and “Area of Thessaly Plain, GR1420011”), and an SCI (“Karla–Mavrovouni–Kefalovryso Velestinou–Neochori, GR1420004”) underscoring the area’s ecological significance. The wetland hosts a diverse range of herpetofauna species, encompassing a total of 27 species of which five are anuran amphibians and 22 are reptile species [58].

### 2.2. Mapping and Analysis of Road Network Features

To thoroughly document all instances of herpetofauna species impacted by road mortality in Lake Karla, an accurate and complete mapping of the wetland’s road network was imperative. We achieved this by integrating open-source geospatial data from the Greek platform for geospatial data and services, Geodata.gov.gr (accessed on 10 September 2023), along with proprietary data sourced from the Biodiversity Conservation Laboratory at the University of the Aegean.

The primary road network in our study area serves as a vital hub, intricately interconnecting essential infrastructures and establishing seamless linkages between pumping stations, supply channels, agricultural lands, and urban areas. Encompassing 28 settlements (27 villages and the city of Larissa), these urban interfaces serve as significant connectors, bridging the rural and urban landscapes. The extensive roadways, categorized into main arteries, secondary routes, and dirt roads, cover a substantial expanse of 1556.88 km, with an average width of 7.07 ± 1.85 m. The main arteries, constituting the primary road network, extend over 1020.25 km, forming connections between villages and Larissa. These arteries feature two traffic lanes flowing in opposite directions, complemented by an asphalt hard shoulder with an average width of 7.62 ± 4.33 m. Secondary routes and dirt roads, covering 536.63 km, exhibit an average width of 5.41 ± 2.01 m, linking agricultural areas to critical infrastructures such as pumping stations, supply channels, and villages.

### 2.3. Road Mortality Surveys

We conducted a total of 144 surveys, equivalent to 12 per year, systematically traversing the entire road network of Lake Karla monthly. Each survey spanned three to four continuous days, covering an estimated 350 to 400 km of the road network per day. Furthermore, recognizing the dynamic nature of roadkill events, we adopted a flexible time interval between survey months, ranging from 20 to 30 days. During these surveys, we employed a vehicle equipped with flashing emergency lights, maintaining a controlled speed of 30–40 km/h in adherence to the minimum speed limits set by Greek legislation. Two experienced observers conducted road network surveys along the roadsides during daylight hours, utilizing an alternating direction pattern for animal counts only during transit. Each day, we initiated the survey from a new designated point, systematically covering approximately 350 km before resuming from the endpoint the following day, and each month, our surveys commenced from a different starting point. This rotation allowed us to cover every segment of the vast road network with equal effort. To detect road-killed herpetofauna carcasses from the previous night before further impact by vehicular traffic or scavengers, we started monitoring the road network during sunrise, and we finished the monitoring half an hour after sunset. To prevent double-counting or additional roadkills by opportunistic predators, we consistently removed observed carcasses during subsequent surveys. In cases where safety considerations precluded carcass removal, precautions were taken to minimize instances of double counting. We identified road-killed herpetofauna in the field with a high degree of specificity at the lowest taxonomic level during each survey.

We documented specific details for each roadkill occurrence, encompassing GPS coordinates, date, species, roadside habitat, and road width. The diverse roadside habitats were recorded and categorized into seven distinct types: complex cultivation patterns, fruit trees and berry plantations, sclerophyllous vegetation, permanently irrigated land, non-irrigated arable land, wetland vegetation, and urban areas. To gain a comprehensive understanding of the circumstances surrounding each roadkill incident, we also measured the distance from the nearest water source, and noted whether the roadkill was situated within a Natura 2000 area. Recognizing the impact of weather conditions on animal behavior and their interactions with road networks, we systematically gathered meteorological data using a portable weather station. This involved recording temperature (°C), humidity (%), and precipitation (mm) on a daily basis during our fieldwork.

Simultaneously, we deemed it essential to document traffic volume and speed across the entire road network. Due to the absence of raw data, we categorized both of these road-related variables into distinct classes based on a combination of empirical observations, on-site measurements, and data sourced from relevant transport authorities and law enforcement agencies. We thus characterized roads into high (>100 vehicles/h), denoting sections with consistently dense and heavy traffic; medium (50–100 vehicles/h), indicating areas with moderate and balanced traffic flow; and low (<50 vehicles/h), signifying segments characterized by sparse and minimal traffic activity. Similarly, we classified vehicle speeds as high (>80 km/h) for road sections where vehicles consistently traveled at elevated speeds, medium (50–80 km/h) for areas with a moderate and regulated flow of traffic, and low (<50 km/h) for segments where vehicles typically moved at reduced speeds, such as residential zones or areas with specific speed restrictions.

### 2.4. Spatial Analysis of Roadkill Incidents

In examining patterns of reptile and amphibian road mortalities, we spatially analyzed roadkill incidents by employing three-fold widely used methodological approaches, utilizing the Kernel Density Estimation (KDE), the Getis-Ord Gi* statistic, and the Kernel Density Estimation plus method (KDE+).

KDE served as a foundational element in our analytical approach, unraveling spatial patterns at a broad scale. This technique facilitated the computation of roadkill concentration around each cell of the output raster, employing a “kernel” curve centered at individual data points. The curve’s summation yielded an estimate of the overall density of roadkill incidents, affording us insights into the spatial distribution of these occurrences. KDE assigned varying weights to data points based on proximity, creating a smoothed density estimate that vividly highlighted areas of heightened or diminished roadkill density [59].

To enhance the local-scale validation of the KDE findings, we further proceeded with an additional assessment using the Getis-Ord Gi* method. This technique was employed to evaluate spatial autocorrelation and the similarity of values across various locations. Initially, we created a weighted point feature class representing the frequency of roadkill incidents, named “ICOUNT”, using the “Integrate” and “Collect Events” tools available in the ArcGIS 10.7 Toolbox (ESRI Inc., Redlands, CA, USA). These tools were employed to enhance the spatial integrity of datasets, ensuring that features expected to be coincidental were appropriately snapped together, consequently minimizing gaps or overlaps. During this process, we observed a clustering of roadkill occurrences within a specific distance from one another. To address this, we chose to snap features together within a distance of 500 m, a parameter subjectively determined based on our understanding of the data and the road network of Lake Karla.

Thereafter, utilizing the Mapping Clusters tool within the ArcGIS spatial statistics suite, we generated GiZScore maps, offering z-scores and *p*-values. These metrics played a crucial role in determining the statistical significance of the identified clusters [60,61]. Z-scores, indicating the direction and strength of clustering, were instrumental in gauging the intensity of roadkill clusters. A larger z-score denotes a more pronounced clustering, categorizing the area as a hotspot, while a smaller z-score indicates a more intense clustering of low values, designating the region as a coldspot [62,63]. Using the aforementioned methodological approaches, we produced a series of maps to illustrate the outcomes of our analyses. This visual representation covered the entire dataset and its distinct subcategories, encompassing (a) reptiles, (b) amphibians, (c) species with the highest mortality rate, and (e) different seasons.

Ultimately, we utilized the KDE+ method [64] to refine our analysis, focusing solely on the spatial distribution of roadkills along road segments and their respective lengths. This method extends the capabilities of the KDE analysis by introducing statistically significant testing for each cluster. This not only aids in identifying clusters and the contributing roads, but also facilitates their ranking based on significance. Our application of this method aimed to precisely pinpoint road segments with the highest likelihood of roadkill incidents in our study area [64,65,66]. Initially, we segmented the road network, concentrating on the six roads (RD1, RD2, RD3, RD4, RD5, RD6) with the highest roadkill counts. For each roadkill incident, we measured the distance from the start of the road segment and compared it with the total length of the segment. The collected data served as input for the KDE+ software (Available from https://www.kdeplus.cz/en/, accessed on 24 February 2023), and the results were exported in tabular and graphical formats for further analysis. Additionally, we computed two relative metrics, namely, cluster strength and collective risk [67].

### 2.5. Statistical Analysis

To gauge the relative frequency of roadkill events for each species, we calculated a relative Road Mortality Index (RMI) by dividing the number of roadkills for a specific species by the total number of roadkills across all species [68].

We used chi-square tests to investigate relationships between roadkill incidents and a range of environmental and traffic-related variables. The analysis initially focused on determining significant associations between these factors and roadkill occurrences, considering (a) taxonomic groups, which encompassed reptiles and amphibians, (b) taxa categories, such as snakes, lizards, turtles, amphibians, and (c) the five most impacted species. The examined factors included monthly and seasonal variations, main roadside habitats, road category (main or secondary), traffic volume (high, medium, low), traffic speed (high, medium, low), and if the location of each incident was within a Natura 2000 area. In instances involving continuous variables, including road width, temperature, humidity, precipitation, and distance from water, we utilized t-tests (for taxonomic groups) and an analysis of variance (ANOVA) (for taxa categories and the five most frequently road-killed species), followed by the Games-Howell test for multiple comparisons, to evaluate their influence on roadkill incidents. Owing to the restricted sample size of roadkills from mid-autumn to winter (November to February), we chose to exclude them from the analysis.

We also conducted a series of binary logistic regression models to identify factors predicting the presence of roadkills in our study area. Initially, we employed a single model with the dependent variable encompassing both reptiles and amphibians, considering logistic regression’s applicability to presence-only data. This model aimed to analyze the likelihood of road-killed reptiles and amphibians based on explanatory variables, including all the measured environmental and road-related variables.

Furthermore, we developed three distinct logistic regression models to investigate the factors influencing the presence of roadkill among reptiles and amphibians, encompassing both collective and individual taxa-specific analyses. To create a dataset for these models, we randomly generated pseudo-absences in ArcGIS, matching the exact number of roadkills [69] for reptiles and amphibians. These random points were then included in the logistic regression models alongside the actual roadkill data to evaluate the significance and contribution of various factors in predicting the presence of road-killed reptiles and amphibians. We also extracted road-related variables, including roadside habitats, road width, road category, traffic volume, traffic speed, location within a Natura 2000 area, and distance from water. We evaluated the logistic regression models by creating a classification table to assess the alignment between observed and predicted values and to quantify the accuracy of our models. For increased precision, we optimized the predictor variables through a backward stepwise procedure. Nagelkerke’s R^2^ served as a quantitative index to gauge the explanatory power of our models, expressing the proportion of observed variation accounted for by the included variables. Additionally, the Receiver Operating Characteristic (ROC) curve analysis allowed us to thoroughly examine model performance across various sensitivity and specificity thresholds. Finally, we scrutinized the overall significance of our models using the Hosmer-Lemeshow goodness-of-fit test [70], ensuring their statistical adequacy in representing the complexities of road mortality factors.

We performed all statistical analyses utilizing SPSS software (v. 25.0, Armonk, NY, USA: IBM Corp.)

## 3. Results

### 3.1. Reptiles and Amphibians’ Road Mortality

**Figure 2:**
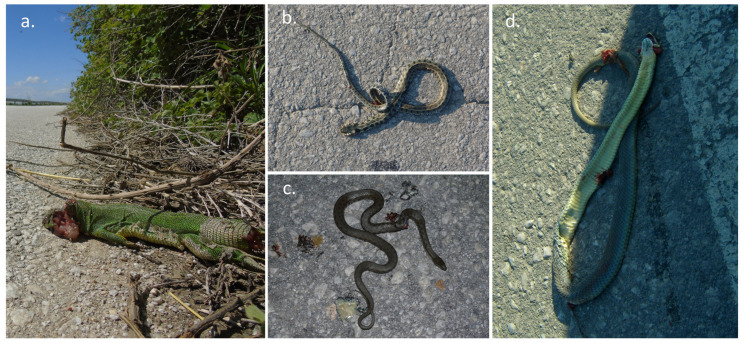
Herpetofauna road fatalities in the main road network of the Lake Karla: (**a**) *Lacerta trilineata*, (**b**) *Natrix natrix*, (**c**) *Natrix tessellata*, and (**d**) *Malpolon insignitus*.

We recorded a total of 340 cases of road mortality involving 14 herpetofauna species, out of the 27 herpetofauna species identified in Lake Karla’s plain. This comprised 280 reptiles and 60 amphibians throughout our observation period from 2008 to 2019 (Figure 2).

The recorded roadkills across diverse taxonomic groups revealed a predominant occurrence of snake fatalities, accounting for 60.29% of the total incidents. Amphibians constituted 17.64% of roadkills, followed closely by lizards at 16.76%, and turtles at 5.29%. Specifically, the snake category encompassed seven distinct species, while amphibians were represented by three species, and both lizards and turtles featured two species each. Among the 14 documented herpetofauna road-killed species, five exhibited higher susceptibility to road mortality: (a) the grass snake (*Natrix natrix*) with 76 instances (RMI = 0.227), (b) the European glass lizard (*Pseudopus apodus*) with 43 occurrences (RMI = 0.126), (c) the Caspian whipsnake (*Dolichophis caspius*) with 38 incidents (RMI = 0.111), (d) the green toad (*Bufotes viridis*) with 33 occurrences (RMI = 0.09), and (e) the Eastern Montpellier snake (*Malpolon insignitus*) with 31 instances (RMI = 0.09). Evaluation of the conservation status of these roadkill-affected species revealed that the majority were classified under the IUCN category of Least Concern (LC). Nonetheless, two species, the European pond turtle (*Emys orbicularis*) and the four-lined snake (*Elaphe quatuorlineata*), were designated as Near Threatened (NT) (Table 1).

Examining road-related factors, the majority of roadkill incidents occurred on roads within the Natura 2000 area, comprising 266 out of the total 340 documented roadkill occurrences over the 12-year observation period. Simultaneously, the primary road network accounted for 72.35% of roadkills, with 147 incidents occurring in high traffic volume areas, 126 in medium traffic, and 67 in low traffic. In terms of traffic speed, 30 incidents occurred in high-speed zones, while 168 and 142 incidents occurred in medium and low-speed areas, respectively.

The environmental and meteorological variables exhibited distinct patterns during the observational span. The diverse roadside habitats were characterized by distinct occurrences, with the permanently irrigated land category registering the highest incidence, documenting 124 individuals. Subsequently, fruit and berry plantations contributed significantly to road mortality, recording 52 instances. The roadkill incidents were noted at varying distances from water sources, with the majority (n = 119) occurring within 100 m, emphasizing the influence of proximity to water bodies on road mortality.

### 3.2. Temporal and Seasonal Trends of Herpetofauna Road Mortality

**Figure 3:**
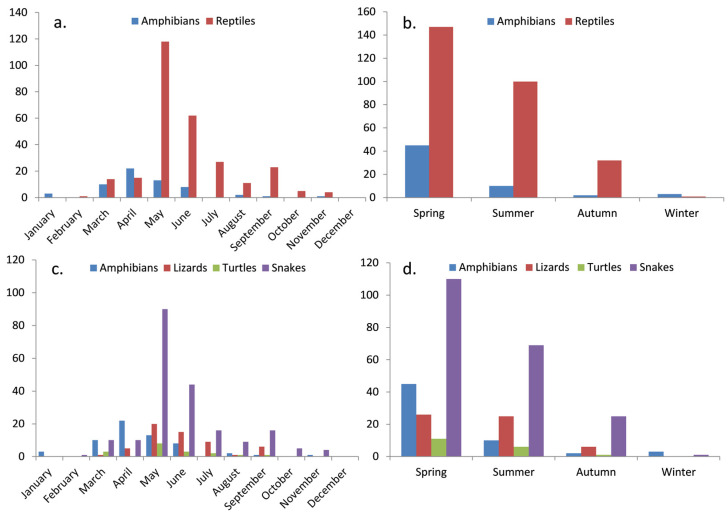
Herpetofauna road mortality incidents distribution, depicting (**a**,**c**) monthly variations and (**b**,**d**) seasonal patterns for both reptiles and amphibians, including the four taxonomic categories.

Seasonal breakdowns revealed distinct variations in road mortality, with certain seasons demonstrating heightened incidents. Spring emerged as the season with the highest roadkill occurrences, constituting 56.47% of the total incidents (reptiles = 43.23%, amphibians = 13.23%) followed by summer with a total of 32.35% (reptiles = 29.41%, amphibians = 0.02%). May and June stood out as the months with the highest reptile roadkill incidents, recording 118 and 62 cases, respectively, while April and May were peak months for amphibians, with 22 and 13 incidents, respectively (Figure 3). In contrast, roadkill incidents involving all categories were scarce throughout the winter months (Figure 3), attributed to the prevailing low temperatures that rendered these species inactive during this period.

Regarding herpetofauna categories, snakes exhibited dominance in spring, summer, and autumn, comprising 32.35% of total roadkill incidents in spring, 20.29% in summer, and 7.3% in autumn. Lizards accounted for 7.35% in summer, while turtles were the least affected category, contributing 5.29% across all seasons (Figure 3).

In terms of species, the grass snake emerged as the most affected, contributing 12.35% of incidents in spring, 7.94% in summer, and 1.76% in autumn. This was followed by the European glass lizard with 5.58% in spring and 5.29% in summer occurrences, and the Caspian whipsnake with 5.58% in spring and 4.11% in summer.

### 3.3. Associations between Herpetofauna Road Mortality and Its Influencing Factors

The chi-square analysis underscored the considerable impact of months (χ^2^ = 71.56, df = 6, *p* < 0.001), seasons (χ^2^ = 13.51, df = 2, *p* < 0.001), traffic volume (χ^2^ = 11.51, df = 2, *p* = 0.003), and roadside habitats (χ^2^ = 12.45, df = 6, *p* = 0.049) on the distribution of roadkills between reptiles and amphibians. These trends extended consistently to the herpetofauna categories, revealing significant variations in months (χ^2^ = 83.39, df = 18, *p* < 0.0001), seasons (χ^2^ = 16.49, df = 6, *p* = 0.011), traffic volume (χ^2^ = 37.79, df = 6, *p* < 0.001), and roadside habitats (χ^2^ = 38.87, df = 18, *p* = 0.003). A distinctive finding was evident in road categories (main and secondary), presenting a significant difference compared to the reptiles and amphibians categorization (χ^2^ = 22.95, df = 3, *p* < 0.001). Assessing the five most frequently road-killed species yielded slightly divergent outcomes, with the majority aligning with herpetofauna category patterns. Traffic speed demonstrated a significant effect on roadkill incidents (χ^2^ = 18.09, df = 8, *p* = 0.021), deviating from consistent trends in other factors. Nevertheless, substantial variations were also identified in months (χ^2^ = 85.68, df = 24, *p* < 0.001), seasons (χ^2^ = 19.68, df = 8, *p* = 0.012), traffic volume (χ^2^ = 38.33, df = 8, *p* < 0.0001), road category (χ^2^ = 17.09, df = 4, *p* < 0.002), and roadside habitats (χ^2^ = 54.09, df = 24, *p* < 0.001).

Contrarily, (a) the distinction between main and secondary roads (*p* = 0.612), (b) the roads located within a Natura 2000 area (*p* = 0.312), and (c) the traffic speed (*p* = 0.119) appear to exhibit no discernible impact on reptiles and amphibians. Likewise, nearly identical factors show no substantial effect on herpetofauna categories and the five most impacted species: (a) roads situated within a Natura 2000 area (herpetofauna categories: *p* = 0.399; most impacted species: *p* = 0.124), and (b) traffic speed (herpetofauna categories: *p* = 0.102).

Analysis of continuous variables, encompassing road width, temperature, humidity, precipitation, and distance from water, indicated significant differences between reptiles and amphibians in nearly all instances. Significant disparities were observed in road width [t (338) = −3.553, *p* < 0.001], temperature [t (338) −5.615, *p* < 0.001), humidity [t (338) = −5.417, *p* < 0.001], and precipitation [t (338) = −2.784, *p* < 0.01] between the two groups, while the comparison of distance from water indicated no statistically significant variation [t (338) = 0.237, *p* = 0.813]. Significant variations were also found between the examined herpetofauna categories as the analysis of variance indicated in (a) road width [F (3) = 4.42, *p* = 0.005], (b) temperature [F (3) = 11.06, *p* < 0.001], (c) humidity [F (3) = 10.58, *p* < 0.001], and (d) precipitation [F (3) = 2.88, *p* = 0.036]. Subsequent post hoc Games-Howell tests indicated that the road width in which the snakes were found (7.28 ± 2.14 m) significantly differed from the amphibians (6.31 ± 0.78 m; *p* = 0.002, 95% C.I. = 0.27, 1.65). Regarding temperature, amphibians (16.45 ± 4.85 °C) differed from both lizards (20.29 ± 4.05 °C; *p* < 0.001, 95% C.I. = −5.90, −1.77) and snakes (19.89 ± 4.14 °C; *p* < 0.001, 95% C.I. = −5.08, −1.80). In terms of humidity, amphibians (42.73 ± 31.36%) differed from lizards (56.74 ± 26.47%; *p* = 0.007, 95% C.I. = −25.08, −2.93), turtles (64.78 ± 17.91%; *p* = 0.003, 95% C.I. = −38.13, −5.95), and snakes (61.32 ± 19.53%; *p* < 0.001, 95% C.I. = −27.37, −9.80). Finally, in terms of precipitation, snakes (1.34 ± 3.74 mm) significantly differed from amphibians (0.03 ± 0.29 m) (*p* = 0.042, 95% C.I. = −0.04, 2.65). In alignment with the herpetofauna categories, the most impacted species exhibited variations concerning their distance from water [F (4) = 4.16, *p* = 0.003]. Following post hoc Games-Howell tests scrutinized specific differences among the species. Regarding distance from water, the Eastern Montpellier snake demonstrated a statistically significant distinction from the grass snake (*p* < 0.05) and the European glass lizard (*p* < 0.05). It is important to note that the green toad was excluded from this analysis due to its predominantly nocturnal activity, which could introduce confounding factors when compared to the other four most impacted species.

### 3.4. Predicting the Occurrence of Herpetofauna Road Mortality

The logistic regression analysis examining the presence of reptile and amphibian roadkill, encompassing a comprehensive set of environmental- and road-related factors, revealed a statistically significant model [χ^2^ (20, N = 340) = 120.1, *p* < 0.05]. This implies that variables such as month, road features, traffic speed, roadside habitats, and precipitation exerted substantial influence in predicting the occurrence of road-killed reptiles and amphibians (Table 2). The model exhibited an overall classification accuracy of 81.2%, with specific accuracies of 82.5% for reptiles and 75% for amphibians, while it achieved an area under the curve (AUC) of 0.874 (S.E. = 0.02, 95% CI 0.82–0.92, *p* < 0.0001), signifying excellent predictive performance. Nagelkerke R^2^ accounted for 47.2% of the total variance in the data, while the Hosmer-Lemeshow goodness-of-fit test produced a satisfactory result (Hosmer-Lemeshow = 8.94, *p* > 0.05).

Moreover, the logistic model, incorporating both actual roadkills and pseudo-absences (where the number of pseudo-absences for reptiles equalled the number of reptile presences, and a similar principle was applied to amphibians), exhibited statistical significance [χ^2^ (6, N = 680) = 350.37; *p* < 0.0001], emphasizing the consideration of exclusively road- and habitat-related variables in this analysis. The reptiles and amphibians model (Table 3a) yielded an overall classification accuracy of 81.3%; 80% for reptiles and 82.6% for amphibians. The Nagelkerke R^2^ value was 0.537, indicating that this model explained a substantial portion of the total variance in the data. The Hosmer-Lemeshow goodness-of-fit test result was 14.65 (*p* > 0.05), suggesting a good fit of the model to the data. Additionally, the AUC for this model was 0.873 (S.E. = 0.01, 95% CI 0.84–0.90, *p* < 0.0001), which signifies a reasonably high level of predictive accuracy.

Examining the logistic model exclusively dedicated to reptiles (Table 3b), a statistically significant model was also evident [χ^2^ (12, N = 560) = 282.48, *p* < 0.0001]. Collectively, variables explained 52.8% of the total variance (Nagelkerke R^2^ = 0.528), while the model demonstrated a well-fitted nature (Hosmer-Lemeshow = 10.75; *p* > 0.05). The AUC (0.87, S.E. = 0.01, 95% CI 0.84–0.90, *p* < 0.05) revealed accurate classification of road-killed reptiles at 81.6%.

Regarding the logistic model that incorporated only the amphibians (Table 3c), we achieved a significant model [χ^2^ (5, N = 120) = 98.57, *p* < 0.05]. The variables traffic volume, traffic speed, and distance from water source explained 74.7% of the total variance in the data. Additionally, the Hosmer-Lemeshow test confirmed the model’s fitness, as the absence of significant chi-square values attested to its acceptability (Hosmer-Lemeshow = 6.73; *p* > 0.05). Furthermore, the AUC (AUC = 0.93, S.E. = 0.02, 95% CI 0.88–0.97, *p* < 0.05) was used to gauge the sensitivity of specificity values and correctly classified road-killed amphibians in 84.2% of cases.

### 3.5. Spatial Clustering and Hotspot Areas of Herpetofauna Road Mortality

**Figure 4:**
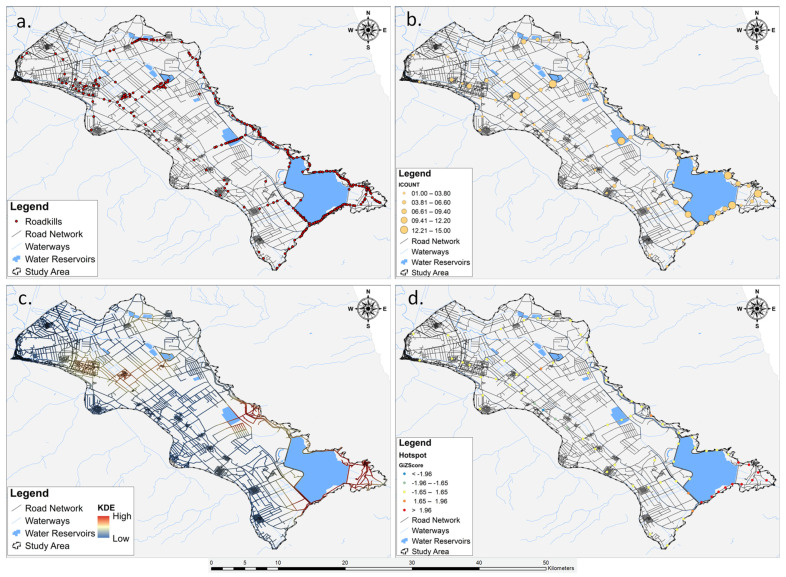
Workflow of the spatial analysis involving multiple steps: (**a**) mapping the distribution of road mortality incidents for herpetofauna species, (**b**) results from the Collect Events analysis, (**c**) visual depiction of road-killed reptiles and amphibians using Kernel Density Estimation (KDE), and (**d**) pinpointing hotspots in the identified areas.

The application of KDE to roadkill incidents provided a spatially detailed representation of reptile and amphibian mortality patterns. The KDE, by generating density surfaces, highlighted areas with concentrated roadkill occurrences across the Lake Karla plain (Figure 4). The resulting maps visually identified hotspots, revealing spatial clusters of elevated densities of road-killed reptiles and amphibians, compared to their surrounding areas, for both taxonomic groups as well as for (a) reptiles, (b) amphibians, (c) five most impacted species, and (e) different seasons.

Results of the hotspot analysis for all the roadkill incidents, conducted using Getis-Ord Gi* statistics, revealed substantial spatial clusters of herpetofauna road mortality hotspots and coldspots. The identified hotspots (n = 11) (Figure 4) displayed z-scores exceeding 1.96, indicating high significance. These clusters are concentrated in the south-eastern part of the Lake Karla plain, encompassing areas such as the restored Lake Karla, a site of ecological rehabilitation efforts which is within the Special Protection Area (SPA) designated as Reservoirs of Former Lake Karla (GR1430007). The z-scores falling within the range of 1.65 to 1.96 (n = 4) indicate a moderate level of significance in the context of the herpetofauna road mortality analysis. These values are distributed across the entire study area, encompassing different geographical regions. Specifically, one z-score is located in the northern part, another in the eastern section, and two in the southern region. Z-scores ranging from −1.65 to 1.65 (n = 57) indicated a lack of significant spatial clustering in herpetofauna road mortality incidents. These scores covered a substantial portion of the study area, suggesting that observed road mortality incidents for herpetofauna in these locations did not significantly deviate from the expected pattern under spatial randomness. The moderate coldspots, characterized by z-scores falling within the range of −1.96 to −1.65 (n = 3), were concentrated in the southwest part of the study area. Additionally, there was one single coldspot (n = 1), positioned amidst the three moderate coldspots in the southwest part of the Karla plain. With a negative z-score lower than −1.96, this specific area suggested a spatial clustering of road mortality occurrences below what would be anticipated by chance.

**Figure 5:**
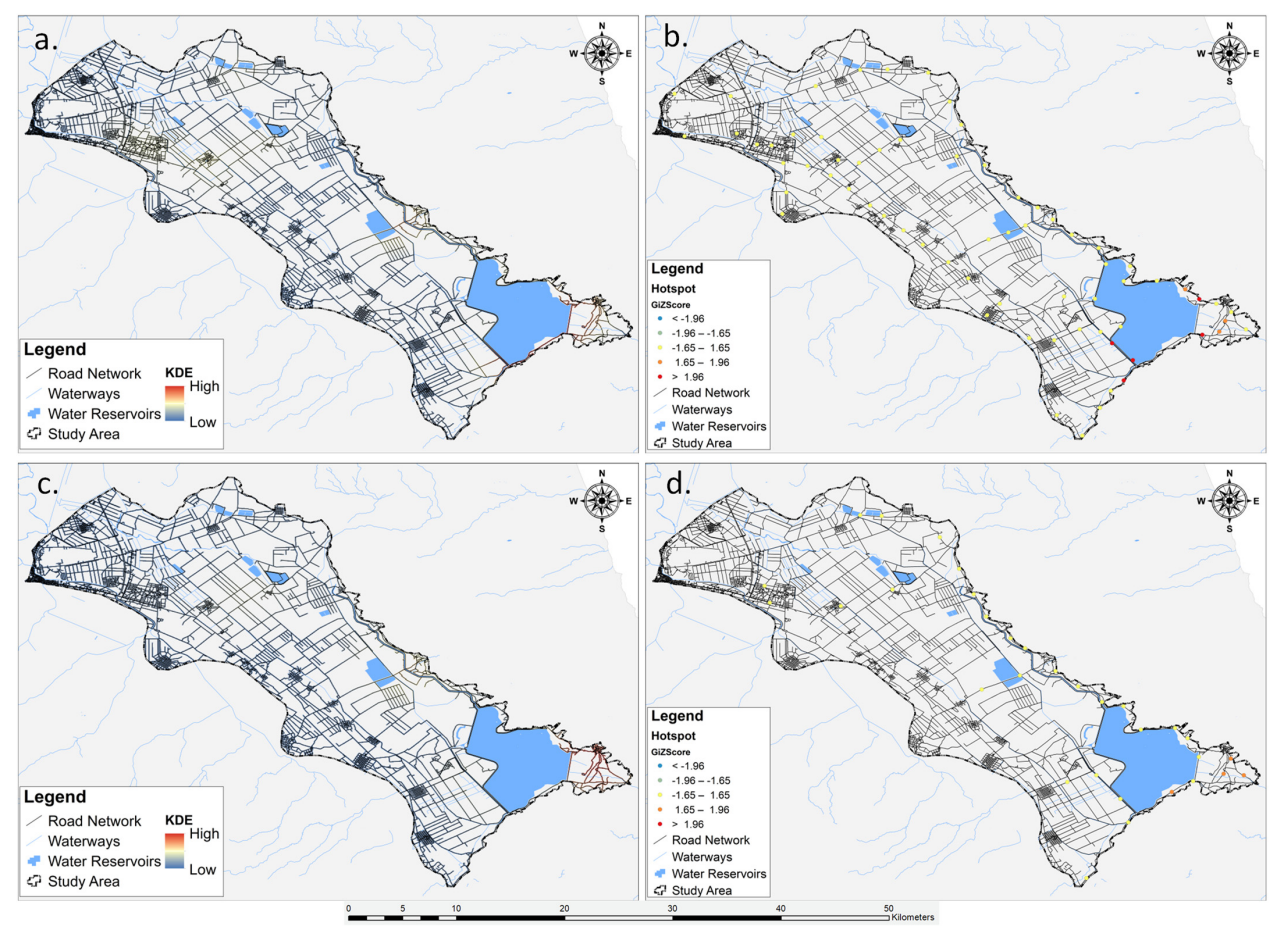
Integration of Kernel Density Estimation (KDE) and Getis-Ord Gi* analysis, illustrating the spatial distribution of roadkill hotspots for (**a**,**b**) reptiles and (**c**,**d**) amphibians.

In the context of reptiles and amphibians, the hotspot analysis revealed distinct patterns in both the spatial occurrence and the degree of intensity of roadkill incidents. Specifically, reptiles exhibited five hotspots and three moderate hotspots, with no identified coldspots or moderate coldspots, as illustrated in Figure 5a,b. On the other hand, despite the KDE for amphibians indicating clustered roadkills along specific roads, the hotspot analysis identified four moderate hotspots, as depicted in Figure 5c,d.

**Figure 6:**
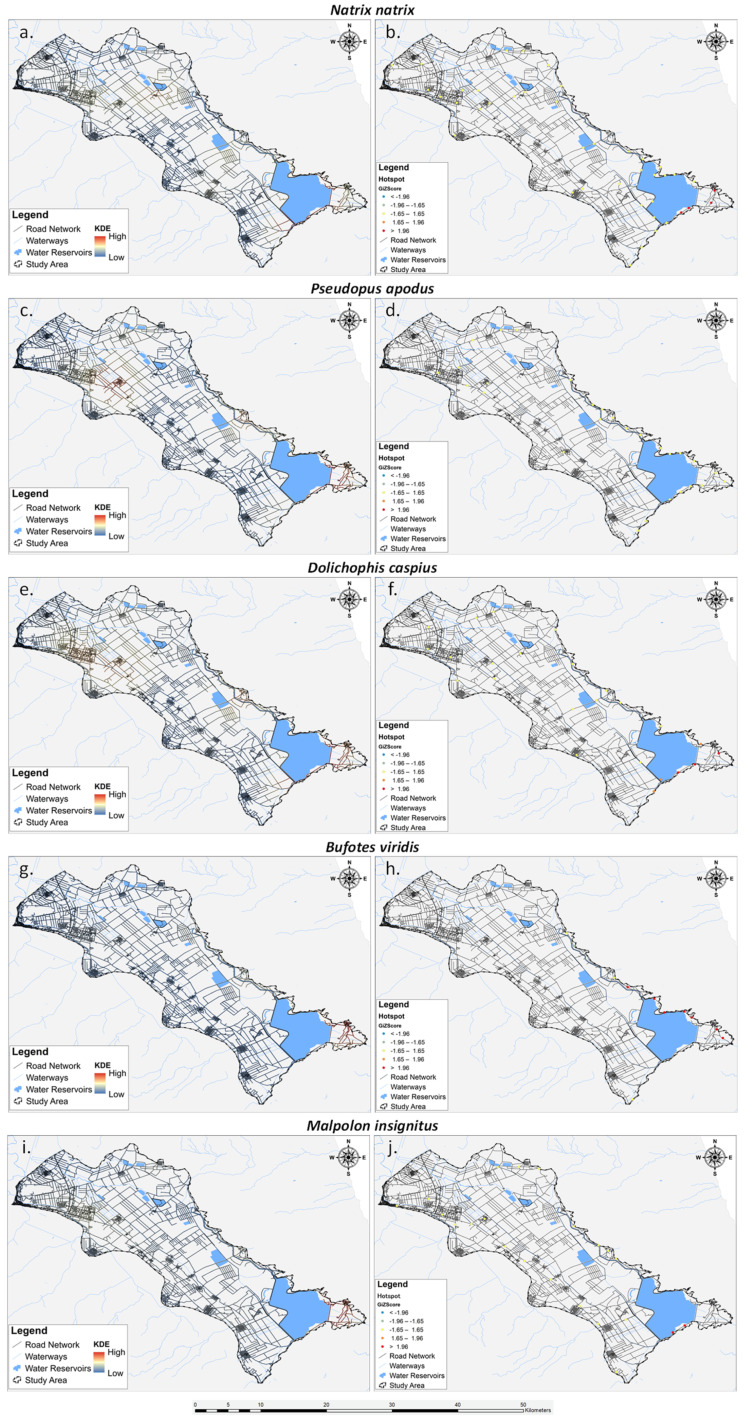
Spatial distribution of the five most road-killed herpetofauna species depicted through Kernel Density Estimation (KDE) (**a**,**c**,**d**,**e**,**g**,**i**) and Getis-Ord Gi* (**b**,**d**,**f**,**h**,**j**).

The analysis of the five most impacted species by roadkills is detailed in Figure 6. All of them exhibit hotspots concentrated in the south-eastern part of Lake Karla. The green toad stands out with the highest number of hotspots, totaling seven, followed by the grass snake with four hotspots. The Caspian whipsnake shows three hotspots and an additional three moderate hotspots, while the Eastern Montpellier snake has two hotspots. Interestingly, the European glass lizard does not exhibit any hotspots in the analyzed area.

**Figure 7:**
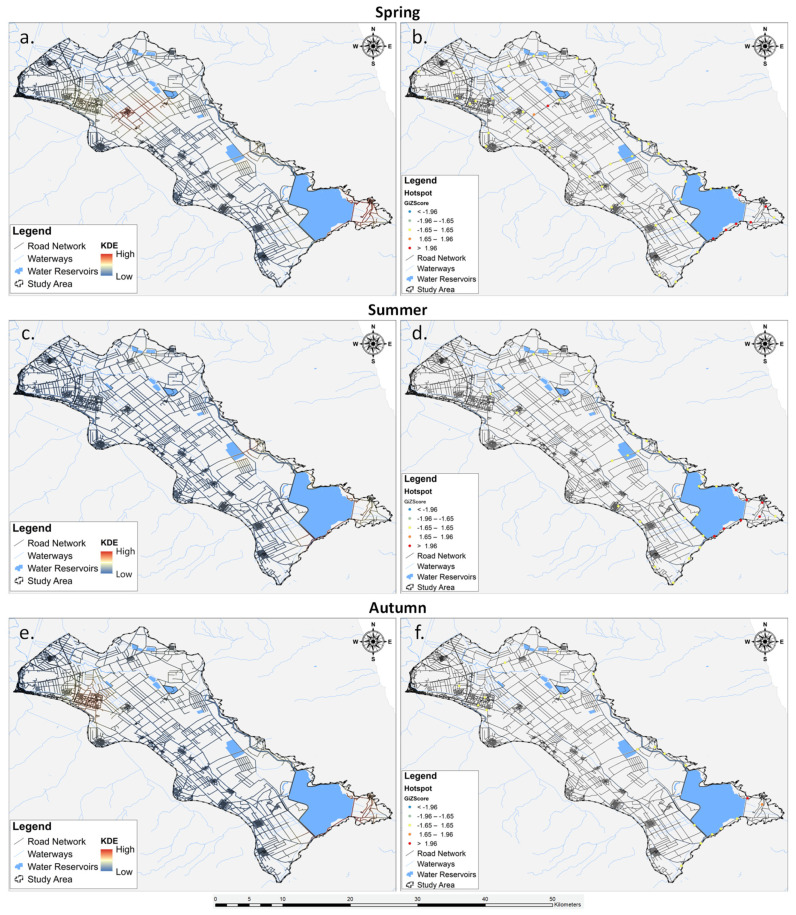
Spatial distribution of the roadkills during seasons (**a**,**b**) spring, (**c**,**d**) summer, and (**e**,**f**) autumn). The analysis for winter was omitted due to the limited raw data.

The seasonal analysis results utilizing KDE and Getis-Ord Gi* are depicted in Figure 7, revealing a slight variation in roadkill hotspots and coldspots across different seasons. It is noteworthy that the analysis for winter was omitted due to the restricted sample size of available data during this season.

During spring (Figure 7a), the KDE showcased spatial clusters of roadkill hotspots concentrated in the south-eastern part of the Lake Karla plain. These hotspots (n = 7) were further confirmed as statistically significant by the Getis-Ord Gi* analysis, with z-scores surpassing 1.96, indicating a high degree of spatial clustering. Moving into summer (Figure 7b), the KDE highlighted roadkill hotspots primarily in the same south-eastern region observed during spring. The hotspots remained consistent between the two seasons. Lastly, in autumn (Figure 7c), the KDE revealed a single hotspot in the south-eastern part of the study area.

### 3.6. Identification of Significant Clusters and Ranking Road Segments

**Figure 8:**
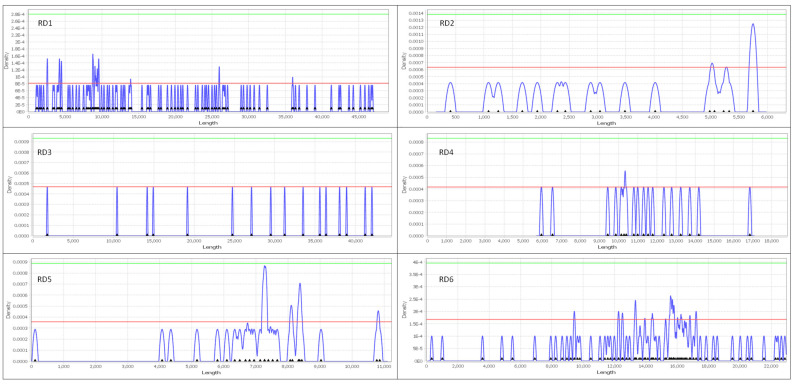
Visual depiction of the six analyzed road sections (RD1–RD6). The X-axis represents the total length of each road section, and the Y-axis represents the density function. The horizontal red line denotes the 95th percentile level. Significant clusters (risk locations) are identified where the blue line surpasses the red line, while the remaining clusters are not statistically significant.

The KDE+ analysis showed that from the six road sections (RD1, RD2, RD3, RD4, RD5, RD6), a total of 28 significant clusters were identified (Figure 8) encompassing 74.11% of the roadkill incidents, while the remaining roadkills did not form significant clusters. These sections exhibited an average length of 25.47 ± 17.42 km, with the clusters having a mean length of 140.79 ± 116.18 m constituting 0.25% of the total road network. The density of roadkills within the clusters, expressed per 100 m, amounted to 2.19 ± 0.55. Detailed information, including the length of each road section, the number of roadkills, the number of clusters and their mean length, and the mean density of roadkills in these sections are presented in Table 4 and in Figure 8.

**Figure 9:**
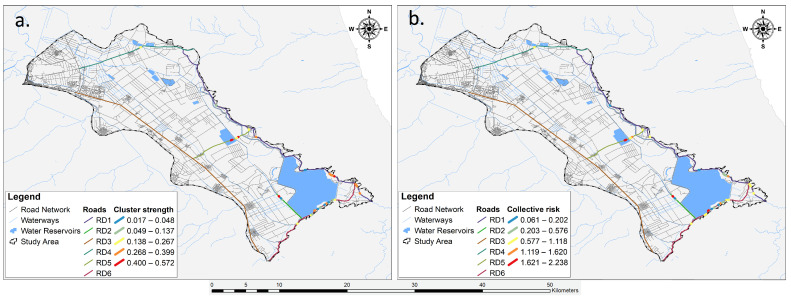
Representation of the six analyzed road sections (RD1–RD6) depicting: (**a**) segments of each road section with the highest likelihood of roadkill incidents (cluster strength) and (**b**) segments of each road section indicating the overall nature of the road (collective risk).

Additionally, the cluster strength exhibited a mean of 0.25 ± 0.17, while the collective risk demonstrated a mean of 1.05 ± 0.70. Specifically, the cluster with the highest statistical significance concerning individual risk (cluster strength) was identified in section RD5 (cluster strength = 0.572, cluster length = 253.066 m, roadkill density = 1.976, collective risk = 2.102). This was followed by a cluster in section RD1 (cluster strength = 0.504, cluster length = 173.148 m, roadkill density = 1.732, collective risk = 1.514) and another cluster again from section RD5 (cluster strength = 0.494, cluster length = 150 m, roadkill density = 2, collective risk = 1.976). Conversely, the clusters with the highest statistical significance concerning sections with the highest collective risks were located in RD2 (cluster strength = 0.493, cluster length = 130 m, roadkill density = 2.308, collective risk = 2.238), followed by a cluster in section RD6 (cluster strength = 0.314, cluster length = 106.871 m, roadkill density = 2.857, collective risk = 1.997). Remarkably, the cluster in RD5, which had the highest individual risk, also featured in a prominent rank among the sections with the highest collective risks (Figure 8). The road sections with all the information regarding the cluster strength and the collective risk are presented in Figure 9.

## 4. Discussion

In this study, we presented a holistic and multidimensional approach to investigate and evaluate the complicated concept of herpetofauna road mortality within the unique ecological context of the Lake Karla plain. Our emphasis on PAs, and especially a restored wetland complex such as Lake Karla, is of high importance considering its unique ecological characteristics and the substantial ecosystem services value that it offers [71,72]. However, persistent conservation challenges, particularly from roads, contribute significantly to ecological disruptions within PAs [73,74], especially in transition zones between terrestrial and aquatic areas [35,75]. This interference poses a heightened risk to fauna, making roads around wetlands perilous for a broad spectrum of taxa, including reptiles [76,77] and amphibians [78,79].

With restored wetlands gaining global conservation attention, understanding the implications of road-related threats in these areas becomes crucial for effective management and mitigation strategies [80]. In alignment with this, our 12-year monitoring period represents a significant milestone in the temporal dimension, constituting the first extensive and time-continuous monitoring initiative of its kind in Greece. After all, long-term studies are imperative for capturing temporal variations in roadkill patterns, enabling the identification of trends, potential contributing factors, and the development of targeted conservation measures [14,81].

However, notwithstanding our extensive monitoring efforts and the employment of a methodological approach involving two proficient observers, it is imperative to refrain from interpreting our findings as conclusive evidence of species population endangerment. The inherent limitations of our study include the likelihood that certain fatalities evaded documentation, resulting in an underestimate of the actual number of herpetofauna casualties within Karla’s road network. This underestimation can be ascribed to various factors such as scavenger activity [82,83,84], post-accident mortality of injured individuals [68], the influence of weather conditions [85], road type [86], and detectability, which can be influenced by carcass size [87], the amount of roadside vegetation [88], displacement by traffic [89], and survey method [88]. Regarding survey methodology, our decision to employ a vehicle and adopt a cautious, low-speed driving approach to enhance roadkill detection, though a widely acknowledged sampling method for studying road mortality in reptiles, amphibians, and vertebrates in general [90,91,92], has been previously highlighted for potential underestimation up to 12–16 times less than the actual recorded roadkill count [93]. This might explain the reduced efficiency in detecting smaller herpetofauna species in the study area. Additionally, as our surveys predominantly occurred during the day, the lower amphibian counts could be attributed to their nocturnal activity, contributing to potential underestimation. While more frequent and intensive surveys would enhance precision, especially for small animals [94], we deliberately opted for this approach to cover the entire road network of Lake Karla’s plain, prioritizing a holistic understanding of the issue over accuracy.

Thus, despite the conservative estimate of the total roadkills in the road network of the Lake Karla plain, we recorded more than half (51.85%) of the known herpetofauna species pool from the entire area [58]; three out of five amphibians, two out of seven lizards, two out of five turtles, and seven out of ten snakes. Notably, specific herpetofauna categories and species exhibit varying susceptibility to road-related fatalities. Reptiles, with 82.35% representation, particularly snakes (seven species), and individual snake recordings (60.29%), were identified as more vulnerable to vehicular accidents compared to other categories, aligning with the higher richness of these species in the region. Among the reptiles, the grass snake stands out with the highest recorded mortality rate, reflecting its prevalence in the study area and its extensive ecological range, encompassing both aquatic and terrestrial environments [58,95,96,97]. In a similar context, the European glass lizard secures the top spot in the lizards category and ranks second in both the reptiles taxonomic group and total roadkills, particularly thriving in the adjusted foothills of Mounts Mavrovouni and Megavouni (Figure 1). The Caspian whipsnake, ranking second in the overall snakes category and third within the reptiles taxonomic group and total roadkills, is also significantly affected due to its widespread distribution across the Lake Karla plain [58].

The heightened incidence of roadkill that we observed in reptiles, particularly snakes, can be elucidated by a confluence of ecological and behavioral determinants. The diverse range of habitats along the road verges of Lake Karla’s network, providing extensive ecological niches for snake species [98,99], such as the grass snake and Caspian whipsnake, elevates the likelihood of road encounters. This spatial overlap between their habitats and road networks significantly contributes to escalated encounters and ensuing fatalities. Additionally, their exhibited thermoregulatory behavior accentuates their susceptibility to vehicular accidents [32], while the foraging proclivity of reptile species, particularly those actively utilizing open environments, heightens the probability of utilizing roads or adjacent open vegetation for foraging purposes [99,100]. Diverging from the road avoidance behavior exhibited by small-bodied species, large-bodied species, including snakes, often do not refrain from crossing roads, in general vertically [101,102] resulting in increased mortality rates [103,104]. In our case, three out of seven snake species display a body length exceeding 25% of the road width during crossings on main roads and surpassing 35% when traversing secondary roads vertically. Moreover, 80.29% of the road-killed herpetofauna occurred in high- and medium- traffic volume zones, aligning with the general notion that elevated roadkill rates correlate with moderate and/or high traffic volumes, especially for species with limited road avoidance [105].

Contrary to expectations, amphibians, recognized for their heightened susceptibility to roadkills [29,33,106], surprisingly displayed much lower roadkill records. This unexpected trend can be partially explained by the species’ behavioral attributes, such as their tendency to move with a wider distribution of angles when crossing a road [101], and/or topographical characteristics of roadways intersecting their habitats, especially elevated positions.. For amphibian species such as the green toad, which constitutes the most frequently road-killed amphibian (Table 1) and is abundant in the eastern part of the study area along the Mavrovouni foothills, the need to traverse an elevated road introduces complexity to its migratory pathways (Figure 1). During the breeding season, when these amphibians transition from their habitats to freshwater bodies to meet their basic life history requirements [107,108], the somewhat elevated roadways in the area present potential barriers, influencing migration patterns and possibly diverting them toward alternative routes to access wetlands, thereby reducing the incidence of road-related mortality.

The examination of temporal and seasonal trends in herpetofauna road mortality revealed complex patterns, indicating a peak in roadkills during the last month of spring and the first month of summer (Figure 3), attributable to the phenology, activity patterns, and local climatic conditions influencing herpetofauna behavior [14,49,58,109]. Specifically, the continued intense activity of certain species during the breeding season and the metamorphosis of tadpoles, prompting their movement toward drier habitats, contribute to this observed peak [110]. Furthermore, the rising temperatures during this period facilitate thermoregulation, amplifying herpetofauna activity on roads [32]. The associations observed between herpetofauna road mortality and the impact of months and seasons extend across all three examined categories, encompassing reptiles and amphibians, as well as the herpetofauna categories and the most impacted species. This temporal and seasonal analysis is further amplified by the disparities identified in environmental factors, contributing to our understanding of the observed mortality patterns.

The intricate impact of road-related variables on herpetofauna road mortality introduced a heightened level of complexity into our analytical framework. Across all examined herpetofauna groups, (a) traffic volume, (b) road width, and (c) roadside habitats emerged as significant influencers of roadkill occurrences, aligning with previous studies that have identified these factors as key determinants of roadkill rates [90,105,111,112,113]. Upon closer examination within herpetofauna categories, the type of road also became a crucial factor, as highlighted by variations in roadkill rates associated with road categories in previous studies [111,114]. Furthermore, the analysis of the most impacted species revealed that traffic speed and distance from water exerted additional influences on road mortality. After all, reptiles and amphibians near ponds and wetland sections are greatly affected by road traffic [78] (e.g., mass seasonal road mortality due to animals’ breeding). This is further amplified by the ‘pausing’ behavior observed in some reptiles in response to vehicle proximity which further accentuates increased roadkill rates, as they tend to pause or become immobile upon a vehicle’s approach [101,115].

In light of these findings, the subsequent analysis of environmental and road-related variables aimed to predict reptile and amphibian roadkills provided valuable insights into the complex factors that can predict these incidents. On the one hand, the evaluation of all these variables underscored its effectiveness in accurately determining roadkill prediction probabilities for both taxa groups together, leading to a model with an overall classification accuracy of 81.2%. On the other hand, the incorporation of pseudo-absences served as a strategic approach to enhance predictive accuracy, bolster robustness, and optimize sample size across three comprehensive models (Table 3). This methodological refinement, tailored to each taxonomic group’s specific needs, revealed nuanced patterns in roadkill incidents, laying the foundation for informed conservation strategies. The models, especially the one focused on amphibians, demonstrated high accuracy and explanatory power, emphasizing the effectiveness of specific predictors like traffic volume, speed, and distance from water bodies in predicting amphibian roadkill events.

The spatial distribution patterns of roadkills provided important information into the hotspots and coldspots among the examined herpetofauna categories. However, it is important to note that roadkill locations may not accurately reflect the actual point of impact, given the potential displacement of animals by passing vehicles post-collision. To address this potential discrepancy, where features need to be aligned but suffer from slight misalignment due to data collection or processing errors, we implemented a 500 m snapping process, aligning features within this distance to the nearest road segment. We arrived at this decision after carefully considering the complex road network of Lake Karla’s plain. Interestingly, the practice of utilizing knowledge of the road network to identify optimal distances is not uncommon, as evidenced in previous studies [90,116].

The density surfaces created through KDE to depict the spatial distribution of roadkill incidents exhibited discernible patterns, albeit with subtle variations. Nevertheless, the recurrent concentration of roadkills in the south-eastern part of Lake Karla’s plain underscores the significance of this specific region as a potential hotspot for herpetofauna road mortality. The observed spatial patterns suggest that certain environmental and road-related factors might converge in this area, contributing to an elevated risk of roadkill incidents. Additionally, the distinct variations in spatial patterns among taxonomic groups, most impacted species, and seasons highlight the complexity of the interactions between wildlife movement, environmental factors, and road infrastructure, reinforcing the idea that certain species are particularly susceptible in specific locations [42] while seasonal variations indicated shifts in roadkill hotspots over time [23,46].

Transitioning from KDE to Getis-Ord Gi* analysis enriched our understanding of the roadkill spatial distribution patterns by providing a statistical assessment of clustering and identifying areas of significant hotspots and coldspots. While KDE highlighted general trends and concentration areas [117], Getis-Ord Gi* offered a more rigorous evaluation of whether the observed clustering was statistically significant or merely a result of chance [60]. This statistical significance adds a layer of confidence to our interpretation of roadkill concentration areas, allowing us to distinguish between random spatial occurrences and areas where factors, whether environmental or anthropogenic, contribute to elevated roadkill incidents. The identified roadkill hotspots, in agreement with KDE results, emphasize the south-eastern region of Lake Karla’s plain as a critical area necessitating urgent mitigation measures, substantiated by statistically validated clustering patterns [118]. The importance of these hotspots can be attributed to the area’s high biodiversity. Nestled within the Mavrovouni and Megavouni foothills, this region forms a mosaic of diverse habitats, encompassing natural vegetation, streams, almond groves, arable crops, pastures, and meadows. This habitat diversity provides advantages for herpetofauna, offering refuges and overwintering sites, favorable abiotic conditions, suitable nesting sites, and increased prey availability compared to agricultural lands where chemical pest suppression is prevalent (pers. obs). Furthermore, the lakeshore adjacent to this region features irrigation and drainage channels, ideal for amphibian species. However, the simultaneous presence of these habitats with road segments crossing various ecological zones and fragmenting the entire area, along with the elevated traffic volume, significantly contributes to the concentration of roadkill incidents, particularly leading to a higher rate of reptile roadkill [119]. Regarding seasonal variations in hotspot distribution, the identified hotspots exhibited slight differentiation in both position and frequency across seasons. Notably, spring and summer emerged as the primary periods of concern (Figure 7), attributed to heightened vehicular traffic resulting from increased agricultural activities, given the predominantly rural character of the study area. Additionally, in the summer, there is an influx of visitors contributing to the elevated vehicular traffic in the identified hotspots.

Finally, the application of KDE+ analysis in our study not only contributed meaningful knowledge into the spatial distribution and characteristics of roadkill hotspots along the six road sections that we studied, but also gave us the ability to use two key metrics, cluster strength and collective risk, which play a pivotal role in assessing the risk associated with these segments. By integrating these metrics, our study enhances the precision of risk assessment along the road network, contributing to the development of targeted conservation strategies, and aiding decision-makers in prioritizing mitigation measures and interventions in a manner aligned with their specific conservation goals and priorities. Cluster strength, ranging from 0 to 1, holds significance for drivers as it reflects the individual risk within a specific cluster or area along a particular road section [66]. The higher the value of this index is, the higher the number of roadkill incidents within the cluster. Conversely, collective risk serves as a comprehensive metric that considers both cluster strength and the density of points within a cluster per 100 m [67], thereby offering a significant understanding of the overall peril of the entire road. While collective risk lacks specific categorization ranges, it becomes a valuable tool for inter-segment comparisons in our study (Figure 9). As a result, stakeholders responsible for implementing mitigation strategies possess the flexibility to interpret and prioritize risks according to their specific objectives and priorities [120].

Hence, the 28 significant clusters identified through the study can be ranked based on their cluster strength and collective risk metrics [64]. These metrics provide a quantitative measure of how far the observed pattern of roadkill incidents deviates from the null hypothesis of uniform distribution along a road section. This is the reason why we did not find a single significant cluster in RD3; the roadkills were uniformly distributed along this section. Among the sections where significant clusters were identified (Figure 8 and Figure 9), RD5 and RD1 emerged as particularly crucial concerning individual risk, while RD3, RD6, and RD5 exhibited prominence in collective risk. From these road sections, RD5, exhibiting two high values for both indices in two out of four significant clusters, emerges as particularly pivotal. Thus, targeted interventions, such as the implementation of wildlife crossings [121,122,123] and culverts accompanied with fencing in these road sections [124,125], may prove highly effective in addressing hotspots characterized by elevated individual risk, exemplified by the cluster in section RD5. Conversely, more comprehensive measures like fencing, traffic speed reducers, and signage at hotspots [126] might be better suited for mitigating hotspots associated with heightened collective risk, such as those observed in sections RD2 and RD6.

## 5. Conclusions

In conclusion, our study on herpetofauna road mortality within the unique ecological context of Lake Karla’s plain has contributed valuable insights into the challenges faced by reptiles and amphibians in such ecosystems and broadening the scope of wildlife conservation in Greece, (a) by highlighting the importance of incorporating multi-dimensional approaches for effective management and mitigation strategies in the face of evolving ecological challenges, and (b) by providing a foundation for informed conservation strategies, aiding decision-makers in prioritizing interventions aligned with specific conservation goals.

Despite the inherent limitations, including potential underestimation of roadkill counts, our 12-year monitoring initiative represents a significant milestone in Greece, capturing temporal variations, identifying trends, and revealing nuanced patterns in more than half of the known herpetofauna species pool in the area. Our findings underscore the significance of specific road-related variables such as traffic volume, road width, and roadside habitats, as well as the importance of road segments like main or secondary roads in influencing roadkill occurrences. Moreover, the heightened vulnerability of reptiles, especially snakes, emphasizes the need for targeted conservation measures, considering the complex interplay of ecological and behavioral determinants.

The spatial distribution patterns and hotspot analyses reveal critical areas, particularly in the south-eastern region of Lake Karla’s plain, where urgent mitigation measures are essential. The application of KDE+ analysis, incorporating metrics like cluster strength and collective risk, enhances the precision of risk assessment along the road network. The identified significant clusters, particularly in road sections RD5, RD1, RD3, RD6, and RD2, offer guidance for targeted interventions, including wildlife crossings, culverts, fencing, and traffic speed reducers, to address both individual and collective risks associated with roadkill incidents.

## Figures and Tables

**Table 1:** Checklist of herpetofauna species recorded as roadkills during monitoring surveys on Lake Karla. The number of roadkills regarding each species is provided, along with the Annexes of Council Directive 92/43/EEC and the IUCN Categories corresponding to each species.

Species	Common Name	Roadkills (n)	RMI	92/43/EEC	IUCN
AMPHIBIA (60)					
*Bufo bufo*	Common toad	3	0.008		LC
*Bufotes viridis*	Green toad	33	0.097	IV	LC
*Pelophylax kurtmuelleri*	Balkan frog	24	0.070	V	LC
REPTILIA (280)					
*Emys obricularis*	European pond terrapin	6	0.017	II, IV	NT
*Mauremys rivulata*	Western Caspian terrapin	12	0.035	II, IV	LC
*Lacerta trilineata*	Balkan green lizard	14	0.041	IV	LC
*Pseudopus apodus*	European glass lizard	43	0.126	IV	LC
*Dolichophis caspius*	Caspian whipsnake	38	0.111	IV	LC
*Elaphe quatuorlineata*	Four-lined snake	20	0.058	II, IV	NT
*Zamenis situla*	European ratsnake	8	0.023	II, IV	LC
*Natrix natrix*	Grass snake	76	0.223		LC
*Natrix tessellata*	Dice snake	30	0.088	IV	LC
*Malpolon insignitus*	Eastern Montpellier snake	31	0.091		LC
*Vipera ammodytes*	Nose-horned viper	2	0.005	IV	LC
Total roadkills		340			

**Table 2:** The logistic regression model illustrating the likelihood of roadkill presence for reptiles and amphibians. The Model Log Likelihood serves as an indicator of the model’s fit, and the change in the −2 Log Likelihood measures the improvement in fit compared to a simpler model. Degrees of freedom (df) are employed to calculate the *p*-value and assess the significance of the log likelihood modification. The *p*-value represents the significance level of the change in the −2 Log Likelihood.

Predictor	Model Log Likelihood	Change in −2 Log Likelihood	df	*p*-Value
Month	−135.009	67.883	10	<0.001
Roads	−107.414	12.692	1	<0.001
Roadside habitats	−116.776	31.417	6	<0.001
Precipitation	−108.167	14.199	1	<0.001

**Table 3:** Logistic regression model for the presence probability of roadkills of (**a**) reptiles and amphibians, (**b**) reptiles, (**c**) amphibians. The Model Log Likelihood is a metric of the suitability of the model’s fit, and the alteration in the −2 Log Likelihood gauges the enhancement in fit when compared to a more basic model. The degrees of freedom (df) are utilized to compute the *p*-value, and the significance of the log likelihood modification. The *p*-value embodies the Sig. of the Change, signifying the level of significance of the shift in the −2 Log Likelihood.

Predictor	Model Log Likelihood	Change in −2 Log Likelihood	df	*p*-Value
(a) Reptiles and amphibians				
Road Nature	−309.146	25.990	1	<0.001
Traffic volume	−313.330	34.357	2	<0.001
Traffic speed	−337.003	81.705	2	<0.001
Distance water	−303.437	14.573	1	<0.001
(b) Reptiles				
Road Nature	−255.811	17.779	1	<0.001
Traffic volume	−253.571	13.299	2	<0.001
Traffic speed	−269.571	45.299	2	<0.001
Distance water	−250.736	7.630	1	0.006
Roadside habitats	−254.185	14.527	6	0.024
(c) Amphibians				
Traffic volume	−49.865	31.950	2	<0.001
Traffic speed	−47.623	27.466	2	<0.001
Distance water	−37.313	6.845	1	0.009

**Table 4:** The processed road sections along with their attributes as extracted by the KDE+ software.

Sections	Length (km)	Roadkills (n)	Clusters (n)	Cluster Length (km)	Roadkill Density
RD1	49.025	99	8	1.602	1.607
RD2	6.326	18	3	0.309	2.262
RD3	44.482	16	0	0	0
RD4	18.825	18	1	0.115	1.728
RD5	11.227	26	4	0.584	2.097
RD6	22.970	75	12	1.329	2.641
Total RD1–RD6	152.855	252	28	3.942	1.722

## Data Availability

The data presented in this study are available on request from the corresponding author (Y.G.Z.). The data are not publicly available due to ongoing research efforts.
